# The Relationship Between Work Environment Satisfaction and Retention Intention Among Nursing Administrators in Taiwan

**DOI:** 10.1097/jnr.0000000000000312

**Published:** 2019-09-20

**Authors:** Chiou-Fen LIN, Chung-I HUANG, Che-Ming YANG, Meei-Shiow LU

**Affiliations:** 1 PhD, RN, Professor, College of Nursing, School of Gerontology Health Management, Taipei Medical University, and Adjunct Nursing Consultant, Department of Nursing, Shuang Ho Hospital, Taipei Medical University; 2 PhD, Deputy Director, Center for Management and Development, and Adjunct Assistant Professor, School of Health Care Administration, Taipei Medical University; 3 PhD, Professor, College of Management and College of Humanities and Social Sciences, Taipei Medical University, and Director, Department of Nuclear Medicine, Taipei Medical University Shuang Ho Hospital; 4 MSN, RN, Endowed Professor, College of Nursing, Taipei Medical University, and Chairperson, Board of Controllers, Taiwan Union of Nurses Association.

**Keywords:** nursing administrator, work environment satisfaction, intention to stay

## Abstract

**Background:**

Nursing administrators are essential to ensuring the quality of healthcare provided in hospitals. The nursing manpower shortage that has affected hospitals in Taiwan over the past decade has weighed particularly heavily on nursing administrators, who are expected to maintain high levels of nursing care quality in frequently understaffed healthcare settings.

**Purpose:**

The objective of this study was to explore the relationship between work environment satisfaction and nursing administrator retention in Taiwan.

**Methods:**

This study used a cross-sectional, questionnaire-based survey to collect data from a sample population of nursing administrators. A set of indicators of quality nursing work environments was developed and included in the questionnaire. A total of 1,829 questionnaires were distributed, and the effective response rate was 95.57%.

**Results:**

The average overall rate of satisfaction with the current work environment across all domains was 3.59 (*SD* = 0.61). The highest level of satisfaction was found in the domain of safe practice environment (*M* = 3.83, *SD* = 0.70), and the lowest was found in the domain of informatics (*M* = 3.38, *SD* = 0.91). Length of administrative position tenure was significantly correlated with retention. Each of the eight domains significantly influenced retention. The domain of support and caring was the most significant predictor of nursing administrator retention.

**Conclusions/Implications for Practice:**

Length of administrator position tenure was significantly correlated with nursing administrator retention. Moreover, intention to stay among junior administrators was particularly affected by the support and caring domain. Therefore, it is recommended that nursing departments develop effective strategies to assist and encourage junior administrators to strengthen their career prospects and satisfaction.

## Introduction

The International Council of Nurses has actively promoted the development of positive practice environments since its inception. Positive practice environments are characterized by innovative policy frameworks that focus on recruitment and retention, strategies for continuing education and promotion, adequate employee compensation, recognition programs, sufficient equipment and supplies, and a safe working environment (International Council of Nurses, 2007).

Because of their role in managing the nursing workforce, nursing administrators significantly affect the quality of healthcare provided by hospitals (Dehghani, Nasiriani, & Salimi, 2016). During the past decade, hospitals in Taiwan have been affected by significant nurse manpower shortages and a corresponding decline in the number of nursing administrators (Lin, Lu, & Huang, 2016).

The environment in which nurses practice is extremely hazardous. Nurses face biological, physical, chemical, psychosocial, and ergonomic hazards, among many others. To create a safe practice environment, all of these factors must be taken into account (Ma, Wang, & Chen, 2011; Yildirim & Yildirim, 2007). Kramer and Schmalenberg (2005) suggested that adequate nursing manpower is indicated when sufficient time and manpower are available to provide quality care.

Number of nurses is not a suitable single representative of nursing manpower adequacy. Factors such as workload, work environment, nursing manpower mix, nurse-to-patient ratio, working hours, nursing specialties and skill levels, and ratio of junior nurses must also be considered (Kaur, Kaur, Kalia, & Kumar, 2010). Moreover, nurse salary and welfare must be commensurate with salary and welfare.

In the professional specialization and team collaboration domain, Schmalenberg and Kramer (2008) pointed out that special attention must be paid to five factors that ensure excellent teamwork. These include the elevation of a nurse's professional ability, an organizational culture that cares for its employees and patients, robust professionalism, a framework to resolve conflicts, and the support and dedication of administrative superiors. In addition, the simplification of work and the use of informatics are essential to reduce workload. To this end, all aspects of nursing, including teaching, research, and clinical services, can and should be computerized (Chen, 2009). The literature indicates that providing resources for continuing education may be a significant contributing factor to the retention of senior staff, quality of patient care, and nurse satisfaction with their practice environment (Kramer & Schmalenberg, 2005). In the support and caring domain, collaborative, visible management and shared decision making have been shown to positively impact nurse retention (Force, 2005; Friese, 2005; Huang & Lu, 2015). Holden (2006) asserted that support, appreciation, and respect from superiors also play a critical role in nurse retention. Furthermore, work environment quality and job satisfaction have also been shown to significantly affect retention (Balouch & Hassan, 2014; Holston-Okae, 2017; Markey, Ravenswood, & Webber, 2012).

A research team at the Taiwan Union of Nurses Association (TUNA) developed a literature-based research framework in 2012 to assess the current quality of nursing work environments in hospitals. This framework measures environmental quality using the eight domains of practice environment safety; staff quality and quantity; workload, salary, and welfare; professional specialization and team collaboration; work simplification; informatics; personal growth and professional development; and support and caring. In 2013, further related research expanded the content of each domain to include indicators and scoring standards, which were subsequently published in *The Journal of Nursing Research* in 2016 (Lin et al., 2016).

The demand for nurses is increasing continuously because of Taiwan's rapidly aging population. Although it is known that young and inexperienced nurses rely on experienced nursing administrators to help implement clinical practices, few studies have targeted nursing administrators. Therefore, the objective of this study was to explore the relationship between work environment satisfaction and nursing administrator retention in Taiwan, with the intention of determining which factors predict nursing administrator retention.

## Methods

### Research Subjects

This study used a cross-sectional, questionnaire-based survey to collect data from a sample population consisting of nursing administrators working in Taiwan. The study sample was recruited from among participants in a series of nursing administrator training workshops organized by TUNA in 2016. The workshop participants, including nursing leaders, head nurses, supervisors, and directors, were given the survey at the beginning of each workshop. This study was approved by the joint institutional review board of Taipei Medical University (TMU-JIRB No. N201604013).

### Research Instruments

The questionnaire used in this study included a set of indicators of nursing work environment quality that was developed by the research team based on findings published in the literature (Lin et al., 2016). The validity and reliability of this questionnaire were tested and confirmed. The set of indicators covered eight domains (see Table 1) and 65 items. The eight domains were safe practice environment (16 items); quality and quantity of staff (four items); workload, salary, and welfare (seven items); professional specialization and team collaboration (seven items); work simplification (five items); informatics (five items); personal growth and professional development (nine items); and support and caring (12 items). Level of satisfaction with each domain was measured using a Likert scale that ranged from 1 to 5, with 5 indicating the highest level of satisfaction.

**TABLE 1.**
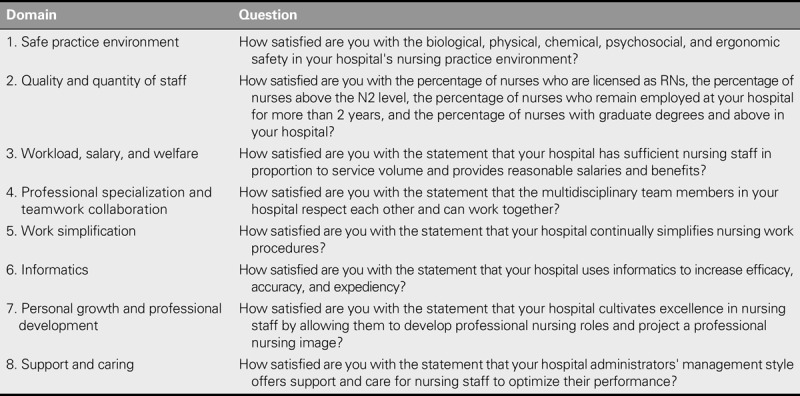
Questions Used to Address Nursing Work Environment Quality

In addition to work environment satisfaction, the questionnaire gathered information on participant characteristics and intention to stay. These characteristics, which were determined in prior studies using expert focus groups (Huang, Yu, & Yu, 2016; Lin et al., 2016), were treated as independent variables and included hospital location, hospital accreditation level, work unit, job position, educational level, duration in administrative positions, nursing seniority, gender, and marital status. Intention to stay was determined by the participant's self-stated willingness to remain employed in the nursing field during the subsequent 3-year period.

### Data Collection and Analysis

The researchers distributed 1,829 questionnaires and retrieved 1,748 effective responses, with a respondent rate of 95.57%. IBM SPSS Statistics Version 19.0 (IBM, Inc., Armonk, NY, USA) was used for statistical analyses, and the significance level was set at *p* < .05. Inferential statistics included the chi-square test, independent *t* test, simple logistic regression, and multiple logistic regression.

## Results

### Participant Characteristics

Data were collected from 1,784 participants. Distributions of participant characteristics are shown in Table 2. The largest number of respondents was from northern Taiwan (*n* = 630, 36.0%). In terms of hospital accreditation level, most (*n* = 855, 48.9%) worked in regional hospitals. Nearly two thirds (31.5%) of participants worked in general wards. In terms of job position, most were head nurses (*n* = 779, 44.6%). Furthermore, most held bachelor's degrees (*n* = 1,091, 62.4%). In terms of duration as a nursing administrator, most had less than a year of experience (*n* = 370, 21.2%). With regard to nursing seniority, most had between 16 and 20 years of nursing practice experience (*n* = 481, 27.50%). Nearly all (98.2%) of the participants were female, and 71.8% were married. Finally, 89.1% indicated that they intended to stay in the nursing field, and only 6.8% (*n* = 118) indicated that they would not stay.

**TABLE 2.**
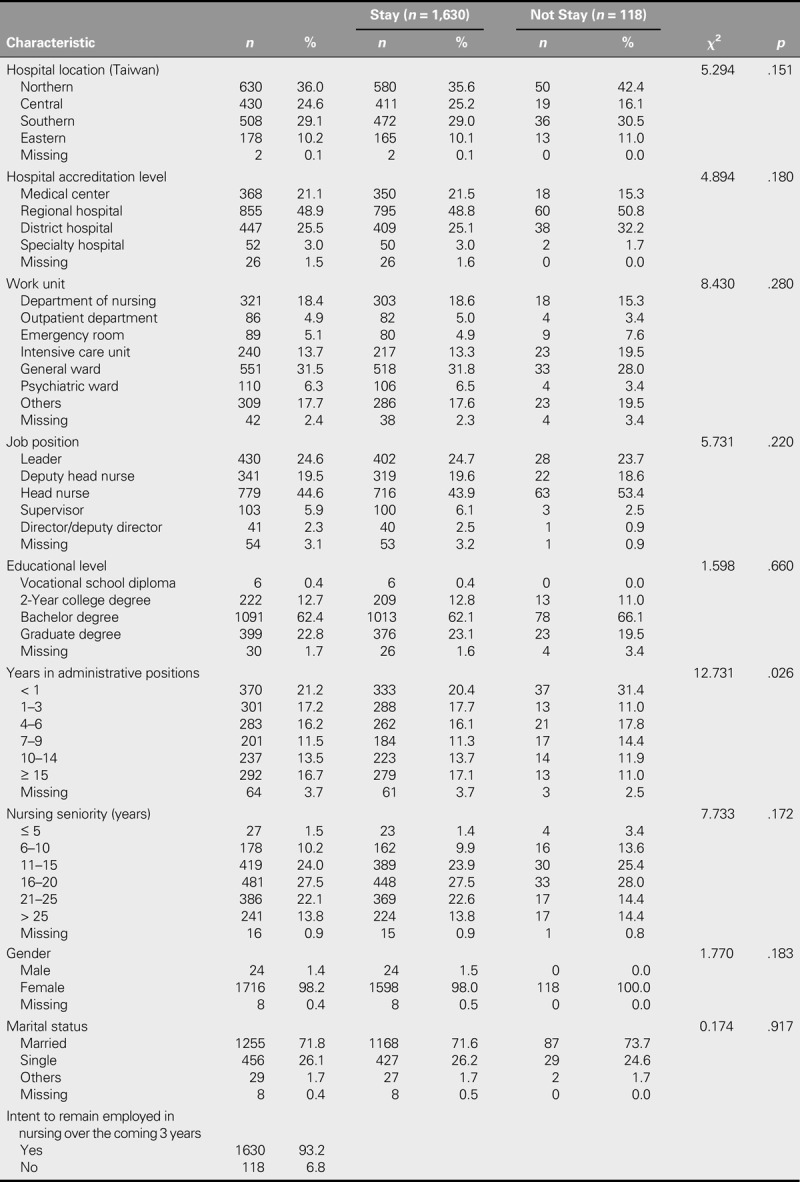
Participant Characteristics and Their Intention to Stay (*N* = 1,748)

Duration of time spent in nursing administrative positions correlated significantly with intention to stay (chi-square test: χ^2^ = 12.731, *df* = 5, *p* = .026). Nursing administrators who had been in administrative positions for less than 1 year reported a lower willingness to remain in the nursing field over the coming 3-year period (*n* = 37, 31.4%).

### Comparative Analyses Between Individual Characteristics and Work Environment Satisfaction

All of the participant characteristics were shown to significantly affect the domains of safe practice environment; workload, salary, and welfare; work simplification; and personal growth and professional development. Similar results were found for hospital accreditation level, job position, and nursing seniority for the professional specialization and team collaboration domain and the support and caring domain. In terms of the quality and quantity of staff domain, hospital accreditation level, educational level, and nursing seniority all showed significant effects. Finally, hospital accreditation level, duration in administrative positions, and nursing seniority all had significant effects on the informatics domain (Table 3).

**TABLE 3.**
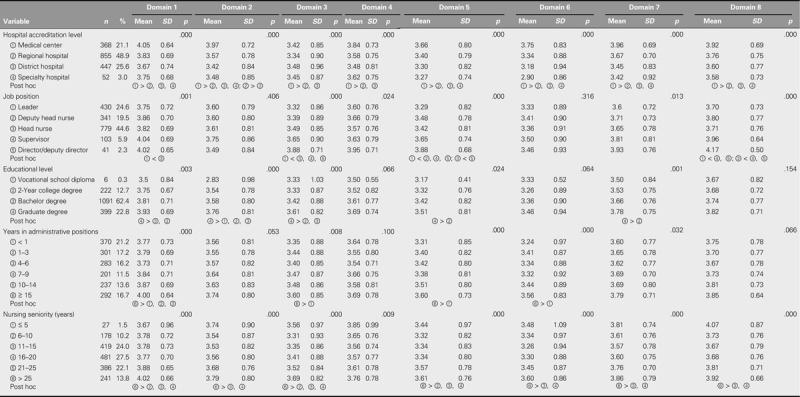
Comparative Analyses of Individual Characteristics and Work Environment Satisfaction (*N* = 1,748)

### Work Environment Satisfaction Scores in Terms of Quality Indicator Domains

The overall satisfaction across all of the eight domains averaged 3.59 (*SD* = 0.61). As detailed in Table 4, the domain of safe practice environment earned the highest average satisfaction score (*M* = 3.83, *SD* = 0.70), followed in descending order by support and caring (*M* = 3.75, *SD* = 0.75); personal growth and professional development (*M* = 3.67, *SD* = 0.76); quality and quantity of staff (*M* = 3.61, *SD* = 0.51); professional specialization and team collaboration (*M* = 3.62, *SD* = 0.77); workload, salary, and welfare (*M* = 3.45, *SD* = 0.87); work simplification (*M* = 3.43, *SD* = 0.81); and informatics (*M* = 3.38, *SD* = 0.91).

**TABLE 4.**
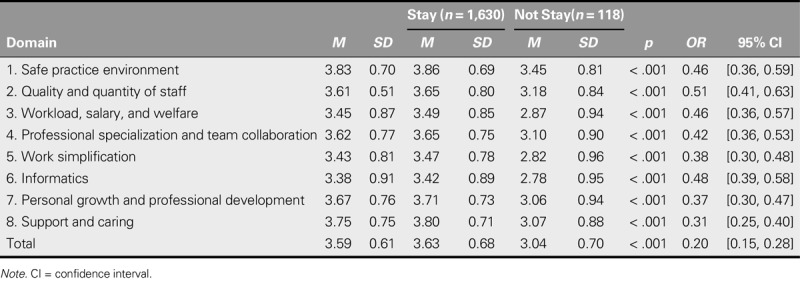
Correlations Between Intention to Stay and Work Environment Satisfaction (*N* = 1,748)

### Correlation Between Intention to Stay and Work Environment Satisfaction

Analysis of data using a simple logistic regression model showed a significant effect of work environment satisfaction on intention to stay. Those participants who intended to stay had an average work environment satisfaction score of 3.63 (*SD* = 0.68), whereas the scores of those who intended to leave averaged 3.04 (*SD* = 0.70). Furthermore, these two groups significantly differed across all of the eight domains (*p* < .001), with the “stay” group scoring higher on each domain than the “leave” group. Significantly, the “leave” group had an average score below 3 on three domains: informatics (*M* = 2.78, *SD* = 0.95); work simplification (*M* = 2.82, *SD* = 0.96); and workload, salary, and welfare (*M* = 2.87, *SD* = 0.946). Univariate analyses showed that all of the domains were valid predictors of intention to stay and that quality and quantity of staff (*OR* = 0.51); informatics (*OR* = 0.48); and workload, salary, and welfare (*OR* = 0.46) were the most significant (Table 4).

### Effects of Duration in Administrative Positions and Work Environment Satisfaction on Intention to Stay

As indicated previously, intention to stay had a significant correlation with the length of time that a nursing administrator had spent in administrative positions. The researchers conducted a multiple logistic regression on intention to stay against duration in administrative positions and each of the other domains of work environment satisfaction. The results indicate that only support and caring achieved statistical significance (Table 5).

**TABLE 5.**
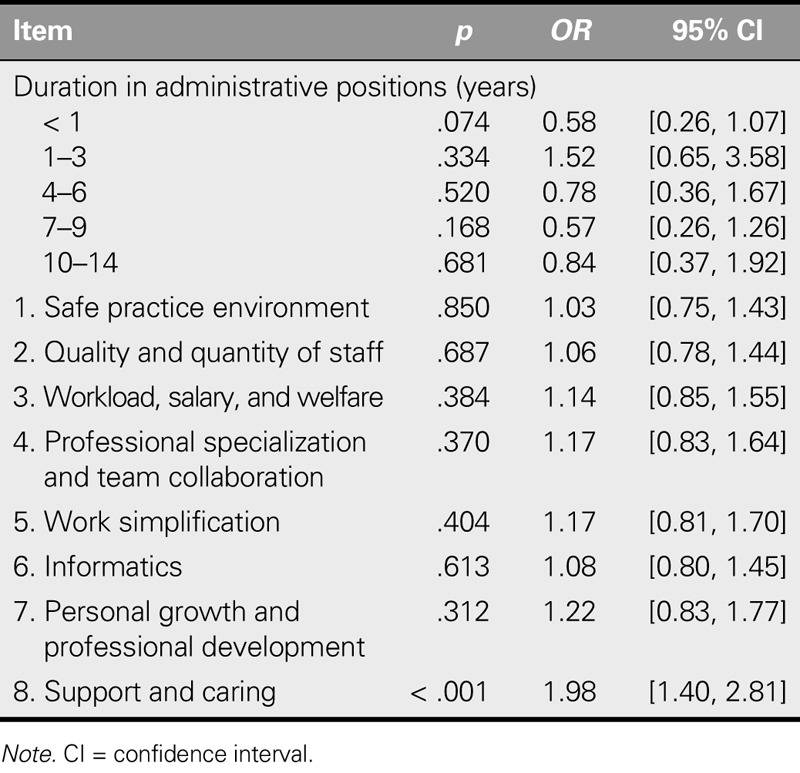
The Effects on Intention to Stay of Duration in Administrative Positions and Work Environment Satisfaction (*N* = 1,748)

## Discussion

The results support the idea that work environment satisfaction is a valid predictor of intention to stay in the nursing field for nursing administrators, which is in line with Warshawsky, Wiggins, and Rayens (2016). In this study, the total overall score for work environment satisfaction averaged 3.59, which is slightly lower than the national average of 3.74, as found in a study conducted in 2013 (Lin et al., 2016). Although the safe practice environment domain reflected the highest level of satisfaction, the average for this domain only reached 3.83, which is significantly lower than the 4.1 obtained in 2013. The nursing administrators who indicated that they would leave the profession in the coming 3 years reported the greatest dissatisfaction in the domains of informatics; work simplification; and workload, salary, and welfare. This result differs from the findings of previous studies. In the previously mentioned 2013 national study, which surveyed all nurses in Taiwan, the three most cited reasons for dissatisfaction were as follows: Salary does not change to reflect work volume, there is a shortage of manpower, and salary is not commensurate with workload. This discrepancy between the current study and the 2013 study may be explained by the different effects of certain factors on retention in nursing administrators and general nurses, respectively. Therefore, the strategies implemented to retain these two tiers of nursing staff should be structured differently.

Nursing administrators expressed dissatisfaction with the informatics domain. There are several possible explanations for this result. First, nursing information systems lag behind other healthcare information systems in terms of development, and many development-related challenges remain unresolved (Hung, 2012). Second, advances in information and communication technologies have affected the process of computerization in ways that have adverse impacts on senior nurses and nursing administrators (Lin & Huang, 2014). Finally, introductions of nursing information systems frequently encounter heavy resistance from nursing staff (Chen, 2011; Gao, 2015; Wu & Hung, 2009).

The results of this study showed that work environment satisfaction differed significantly across hospital accreditation levels, with medical centers earning the highest average score. This result indicates that the work environment in tertiary care hospitals, on average, is superior to those in hospitals of other levels.

Leaders expressed the lowest satisfaction with their administrative jobs. Leaders are the lowest tier of nursing administrators and spend much of their time in direct patient care, which may make their situation psychologically similar to that of general nurses (Chang & Lin, 2016). In terms of educational level, nursing administrators with graduate degrees reported higher levels of satisfaction. The literature offers a possible explanation for this, in that administrators with higher levels of education tend to possess greater problem-solving, leadership, and management abilities (Aiken et al., 2014).

In addition, the results indicate that duration in administrative positions significantly affects intention to stay in the nursing field. In this study, participants with less than a year of administrative experience were more likely to express an intention to leave than their more experienced peers. Nursing administrators face daunting challenges and must grapple with a burgeoning workload that is compounded by personnel shortages (Chang, Lu, & Lin, 2010; Lin, Huang, Kao, & Lu, 2013). Past studies indicate that nursing seniority affects the adoption of strategies. Senior administrators are more likely to adopt positive thinking strategies, whereas junior administrators tend to opt for evasive strategies and to seek support (Chen, Hsu, Yin, Lin, & Chang, 2012; Gellis & Kim, 2004; Shirey, McDaniel, Ebright, Fisher, & Doebbeling, 2010). Therefore, it is imperative to provide adequate leadership and management training for junior nursing administrators to support their adaptation and problem-solving abilities (Dehghani et al., 2016; Lin et al., 2013).

### Conclusions/Implications for Practice

Participants in this study earned an average work environment satisfaction score of 3.59, indicating that there remains significant room for improvement in practice environments and work conditions in hospitals in Taiwan. The eight domains of nursing work environment quality may all be used to predict nursing administrator retention. The informatics domain received the lowest level of satisfaction and thus warrants special attention. In addition, time spent in administrative positions was found to correlate significantly with retention. The domain of support and caring is particularly indicative of intention to stay among junior administrators. Therefore, nursing departments should increase their focus on developing strategies to assist and encourage junior administrators to strengthen the career prospects and satisfaction of these individuals.
